# Tracing of Porcine lncRNA *MALAT1* Shaped by Tissue Localisation and Adipogenesis

**DOI:** 10.3390/genes17080888

**Published:** 2026-07-29

**Authors:** Katarzyna Piórkowska, Ewa Ocłoń, Ksenia Wróblewska, Karolina Zygmunt, Piotr Pawlicki, Laura Pardyak

**Affiliations:** 1 Department of Animal Molecular Biology, National Research Institute of Animal Production, 32-083 Cracow, Poland; ksenia.wroblewska.99.22@gmail.com (K.W.); karolina.zygmunt@iz.edu.pl (K.Z.); 2 Laboratory of Recombinant Proteins Production, Faculty of Veterinary Medicine, University of Agriculture in Kraków, 30-059 Cracow, Poland; ewa.oclon@urk.edu.pl; 3 Department of Infectious Diseases and Public Health, Faculty of Veterinary Medicine, University of Agriculture in Kraków, 30-248 Cracow, Poland; laura.pardyak@urk.edu.pl; 4 Department of Basic Sciences, Faculty of Veterinary Medicine, University of Agriculture in Kraków, 30-248 Cracow, Poland; piotr.pawlicki@urk.edu.pl

**Keywords:** long non-coding RNA, adipogenesis, pigs, preadipocytes, fat deposition, MALAT1, adipose tissue

## Abstract

**Background:** The long non-coding RNA *MALAT1* is known to regulate various cellular processes; however, its role in porcine adipose tissue remains largely unexplored. **Methods:** In this study, we examined *MALAT1* in pigs differing in fat-deposition traits by combining promoter sequencing, in silico analysis of transcription factor binding site analysis, expression profiling, and in vitro adipogenesis assays. **Results:** We identified three novel polymorphisms in the *MALAT1* promoter regions. Several SNPs were predicted to alter binding sites for transcription factors related to metabolism and immunity, such as *NFAT5*, *NFATC1*, and *BCL6B*. One variant, rs329590882, differed significantly in frequency between breeds and may influence *MALAT1* regulation. During adipocyte differentiation, expression of the *MALAT1* isoform ENSSSCT00000080860 increased as adipogenesis progressed. Notably, this isoform was more highly expressed in lean-type Pietrain pigs than in the fatty Złotnicka Spotted (ZS) breed, suggesting that its function may not be directly related to fat accumulation. Additionally, we identified novel exon–exon junctions in *MALAT1*, including a junction associated with the ENSSSCT00000077869 isoform. **Conclusions:** These findings provide new insights into the potential role of *MALAT1* in porcine adipose tissue development and immunometabolic regulation and highlight the need for improved annotation of porcine lncRNAs.

## 1. Introduction

*MALAT1* (Metastasis-Associated Lung Adenocarcinoma Transcript 1) belongs to the class of long non-coding RNAs (lncRNAs), which play crucial roles in epigenetic and transcriptional regulation, including chromatin remodelling [1], microRNA sponging [2], and the enhancement [3] or inhibition of translation [4]. Although lncRNAs were once believed to lack protein-coding potential or to encode only small peptides without biological relevance, recent studies have implicated both lncRNAs and lncRNA-encoded peptides in processes such as carcinogenesis [5] and regulation of immune response [6]. Today, information about peptides encoded by lncRNA show that it plays an important role in the pathogenesis of many diseases [7]. *MALAT1* was initially identified in the context of lung carcinogenesis, as reflected by its name [8]; however, this lncRNA performs a broad range of functions [9,10,11,12]. Studies using patient-derived lung adenocarcinoma cell lines have proven that *MALAT1* overexpression promotes tumour progression and the recruitment of protumourigenic macrophages [13], thereby contributing to the reprogramming of the tumour microenvironment. Moreover, *MALAT1* has been suggested to be an important factor in the development of chemotherapy resistance and, consequently, a potential target for overcoming it [14]. More recently, its role in metabolic disorders, particularly obesity, has been investigated [15,16]. Porcine *MALAT1* was first described by Yang et al. [17], who identified a large set of lncRNAs across multiple porcine tissues. Its presence was subsequently confirmed by Piórkowska et al. [18], who found *MALAT1* expression to be associated with subcutaneous fat (SF) thickness in Złotnicka White (ZW) pigs. Nevertheless, porcine *MALAT1* is still classified as a novel gene in the Ensembl database, and the exact number of its transcript variants has not yet been fully established. According to Ensembl release 113 (October 2024 © EMBL-EBI), porcine *MALAT1* is located on chromosome 2 on the reverse strand at position 6,751,519–6,757,180 and is predicted to have two isoforms, ENSSSCT00000077869 and ENSSSCT00000080860. Promoter regions upstream of both isoforms have been annotated based on ATAC-seq (assay for transposase-accessible chromatin using sequencing) or ChIP-seq (chromatin immunoprecipitation sequencing) data. In our previous study on differentially expressed lncRNAs associated with subcutaneous fat deposition [19], we identified a putative additional *MALAT1* isoform that is not annotated in the Ensembl database. This transcript contains an intronic sequence and exhibits an approximately 84% sequence similarity to human *TALAM1* (Transcript Antisense to *MALAT1*, accession number NR_145459.1), an antisense RNA that overlaps *MALAT1* (NR_002819.5) in the human genome, according to the NCBI.

Adipogenesis is the molecular process by which precursor cells, such as preadipocytes, differentiate into mature, lipid-laden, and insulin-responsive adipocytes. This process comprises several developmental stages, developing from mesenchymal precursors into fully differentiated adipocytes. Successful differentiation requires coordinated changes in preadipocyte morphology, which is regulated by interactions with the surrounding environment and numerous genes that induce or modulate the adipogenic programme. However, the understanding of many molecular aspects of adipogenesis remains incomplete and continues to be refined, particularly in the context of adipocyte turnover and obesity [20]. In addition to excessive caloric intake, obesity is influenced by genetic and epigenetic factors that affect adipose tissue development and function [21]. Increasing attention has also been directed toward non-coding RNA (ncRNA), which exerts a regulatory effect in adipose tissue beyond the protein level. The contribution of ncRNA to adipogenesis has been extensively investigated, particularly in studies focusing on small ncRNA, such as microRNA (miRNA) [22]. More recently, the involvement of lncRNAs in the regulation of adipogenesis and energy metabolism has become increasingly apparent [23]. As ncRNAs may serve as potential therapeutic targets for obesity and related metabolic disorders [24], elucidating their specific functions in adipocyte differentiation is of growing interest. In bovine preadipocytes and differentiated adipocytes cultured in vitro, the lncRNA *ADNCR* (adipocyte differentiation associated long non-coding RNA) was shown to inhibit adipocyte differentiation by acting as a competing endogenous RNA (ceRNA) for miR-204. This interaction increased the expression of sirtuin 1 (SIRT1), a factor associated with the suppression of PPAR-γ activity [25]. In mice, knockout of the lncRNA steroid receptor RNA activator (SRA) was conferred in resistance to high-fat-diet-induced obesity [26]. In our previous study, we identified *MALAT1* as a potential regulator of fat deposition based on its backfat-dependent expression in porcine adipose tissue [18]. However, the reported role of *MALAT1* in obesity and related disorders remains inconsistent [27]. *MALAT1* is highly abundant in exosomes derived from human adipose stem cells and is largely retained during their differentiation into adipocytes [28]. Although *MALAT1* expression decreases in visceral white adipose tissue of aged mice, its function in adipose tissue remains unclear [27]. Other studies have found that *MALAT1* expression in adipose tissue is positively associated with the adipogenesis-related genes *FABP4* and *LPL*, suggesting that *MALAT1* may promote adipose tissue development. *MALAT1* has also be reported to modulate *PPARγ* expression and contribute to adipogenesis through the *PPAR* signalling pathway while also participating in fatty acid metabolism and insulin signalling [29]. In summary, these findings highlight the increasingly recognised functional diversity of lncRNAs in adipogenesis, extending it beyond the regulation of classical protein-coding genes.

Given the limited knowledge of *MALAT1* in pigs, this study aimed to partly address this gap by characterising porcine *MALAT1*, with particular emphasis on its potential role in adipogenesis and fat deposition. To assess its regulatory relevance, we analysed *MALAT1* expression patterns and sequence variants in Złotnicka Spotted (ZS) and Pietrain pigs, which differ phenotypically in their fat-deposition profiles.

## 2. Materials and Methods

### 2.1. Animals

The experimental design included 8 male pigs, with ZS representing the high-fat phenotype (*n* = 4) and Pietrain representing the lead phenotype (*n* = 4). All animals were maintained under standard husbandry and feeding conditions at the Pig Testing Station of the National Research Institute of Animal Production in Krakow, Poland. The pigs were slaughtered at a live body weight of approximately 100 kg. Backfat thickness was measured at the K1 point, located above the lateral edge of the longissimus dorsi muscle. Detailed phenotypic characteristics, including fat- and muscle-related traits, are presented in Table 1 and confirm the phenotypic contrast between the high-fat and lean pig types. Samples were collected from the following four anatomical locations: liver (L), longissimus dorsi muscle (M), abdominal subcutaneous adipose tissue (AD), and backfat, dorsal adipose tissue (AB). All tissues were snap-frozen in liquid nitrogen and stored at −80 °C until further analysis. Additionally, fragments of dorsal subcutaneous adipose tissue measuring 1 × 1 × 0.5 cm^3^ were excised, minced, and placed in depot-specific culture media for primary adipocyte isolation. All animals were used for qPCR and histological analyses. Cell culture experiments, including proliferation and differentiation, as well as Oil Red O staining, were conducted using samples from only ZS pigs. These experiments were performed in three technical replicates, defined as three individual wells per animal on culture plates. Because the animals were slaughtered for food production and tissue collection was performed post mortem, specific approval from the Animal Experimentation Committee was not required. Nonetheless, the experiment was conducted under the supervision and approval of the Approving Experiment Committee of the National Research Institute of Animal Production in accordance with the Polish Act of 15 January 2015 on the Protection of Animals Used for Scientific or Educational Purposes, which implements Directive 2010/63/EU of the European Parliament and Council. All procedures complied with applicable institutional and national ethical standards.

Animal characteristics: The ZS and Pietrain pigs differed significantly in several body composition traits. The two groups showed a similar yield percentage (~75 kg). However, marked differences were observed in fat- and meat-related traits, including ham and tenderloin masses, loin eye area, and subcutaneous fat thickness in the ham, loin, shoulder, and lumbar regions. The greatest differences between the groups were in backfat thickness at the K1 point (cm), located above the lateral edge of the longissimus dorsi muscle. The analysed pig breeds also differed in overall meat and fat contents, which is consistent with previous studies comparing these breeds. Mean values for the selected phenotypic traits are presented in Table 1.

For the analysis of polymorphism in the *MALAT1* promoter region, genomic DNA was isolated from blood samples of 168 pigs, with 24 animals representing each of the following breeds: ZS, ZS, Pietrain, Polish Large White (PLW), Polish Landrace (PL), Duroc and Puławska. All samples were obtained from the biological material collection maintained at the National Research Institute of Animal Production. The samples were previously collected and archived as part of earlier genetic studies conducted at the same institution.

### 2.2. Isolation and Culture of Porcine Preadipocytes

Subcutaneous adipose tissue samples were collected from the dorsal fat depot of adult ZS pigs (*n* = 4). Immediately after excision, 3–4 fragments (~1–2 g) from each depot were aseptically transferred to sterile containers filled with PBS supplemented with 2% Gibco™ Antibiotic–Antimycotic (10,000 units/mL of penicillin, 10,000 μg/mL of streptomycin, and 25 μg/mL of Gibco Amphotericin B, Thermo Fisher Scientific, Waltham, MA, USA). Tissues were manually dissected into smaller fragments under sterile conditions. Preadipocytes were isolated using the Preadipocyte Isolation Kit (Abcam, Cambridge, UK), according to the manufacturer’s protocol, with modifications to optimise for porcine samples. Briefly, fragmented tissue was enzymatically digested using collagenase (1 mL per 0.5 g of tissue) at 37 °C for 30 min with continuous agitation at 150 rpm on a shaker (Corning, Falcon, Sigma-Aldrich, Saint Louis, MO, USA). Digestion was terminated by adding collagenase stop buffer, followed by gentle mixing and filtration through a 100 µm cell strainer (Corning® LSE™ low speed orbital shakers, Corning, Sigma-Aldrich, Saint Louis, MO, USA). The resulting cell suspension was centrifuged at 500× *g* for 10 min, and the supernatant containing mature adipocytes was carefully removed. The pellet was resuspended in 1 mL of Red Blood Cell Lysis Buffer for 1 min, followed by dilution in PBS and filtration through a 70 µm cell strainer. After a second centrifugation, the resulting pellet was resuspended in DMEM/F12 (Gibco, Thermo Fisher Scientific, Waltham, MA, USA), supplemented with 10% foetal bovine serum (FBS; Gibco, Thermo Fisher Scientific, USA) and 1% Gibco™ Antibiotic–Antimycotic (Thermo Fisher Scientific, Waltham, MA, USA), and seeded into 6-well culture plates. Cells were maintained at 37 °C and 5% CO_2_ until reaching 80–90% confluence, with non-adherent cells removed after 24 h.

### 2.3. Materials for Adipogenic Differentiation and Lipid Accumulation Assay

Preadipocytes were induced to undergo adipogenic differentiation using the 3T3-L1 Differentiation Kit (Abcam, Cambridge, UK), following the manufacturer’s protocol. To initiate the process, the culture medium was replaced with fresh medium supplemented with a differentiation cocktail (1 µL/mL; containing insulin, dexamethasone, and IBMX) and cells were incubated for 3 days. After this induction phase, cells were switched to maintenance medium containing insulin (1 µL/mL) and cultured for an additional 7–14 days, with the medium refreshed every 2–3 days to support adipocyte maturation and lipid accumulation. Cells were maintained under standard conditions throughout the culture period.

Adipogenic differentiation was assessed by morphological changes and confirmed using Oil Red O staining on days 2, 7 and 14 to visualise intracellular lipid accumulation. Cells were fixed in 4% paraformaldehyde, stained with Oil Red O working solution, and imaged under a light microscope (Leica, Wetzlar, Germany).

### 2.4. Histological Fat Tissue Preparation

Adipose tissue samples were snap-frozen by immersion in pre-cooled 2-methylbutane (isopentane) chilled with liquid nitrogen to approximately −80 °C. The frozen blocks were stored at −80 °C until sectioning. Prior to cryosectioning, tissue blocks were equilibrated to the cryostat chamber temperature (−20 °C) for 30 min to minimise artefacts. Serial sections of 10 μm thickness were cut using a cryostat (Leica, Wetzlar, Germany) and mounted onto Superfrost Plus microscope slides (Thermo Fisher Scientific Waltham, MA, USA). Sections were air-dried at room temperature for 15 min prior to downstream analyses.

### 2.5. Cell Nuclei Staining of Fat Tissue Cells

Frozen adipose tissue sections (7–10 μm thick) were prepared using a cryostat, air-dried, and fixed in 10% neutral buffered formalin for 10 min. Sections were rinsed in 60% isopropanol and stained with Mayer’s haematoxylin and eosin (H&E) (Sigma-Aldrich, Saint Louis, MO, USA)for 10 min. After staining, slides were washed in 60% isopropanol and distilled water. Sections were mounted with an aqueous mounting medium. Cell nuclei were visualised in blue under light microscopy. Images were acquired using an Olympus (×20) BX51 Upright Brightfield/Darkfield Materials Microscope (Olympus, Tokio, Japan) and processed using ImageJ (National Institutes of Health, Bethesda, MD, USA).

### 2.6. RNA and DNA Isolations

Total RNA was isolated from porcine tissues using protocols adapted to specific tissue types. Subcutaneous abdominal and dorsal adipose was processed with the Tissue RNA Mini Kit (Syngen Biotech, Wrocław, Poland), muscle tissue with TRIsol reagent (Invitrogen, Thermo Fisher Scientific, Waltham, MA, USA), and liver tissue with PureLink™ RNA Mini Kit (Invitrogen, Thermo Fisher Scientific, Waltham, MA, USA), in all cases according to the manufacturers’ instructions. For the in vitro experiment, total RNA was also isolated from preadipocytes at the proliferation stage (day 0) and on days 3, 7, and 14 of adipogenic differentiation using the RNeasy^®^ Micro Kit (Qiagen, Hilden, Germany). RNA integrity (RIN) and quality were assessed using TapeStation 2200 (Agilent Technologies, Santa Clara, CA, USA). Samples were considered acceptable when the RIN was >9 for RNA isolated from cells and RIN > 7 for RNA isolated from tissue.

Genomic DNA was isolated from liver and blood samples collected from pigs representing the following seven breeds: ZS, ZW, Pietrain, PLW, PL, Duroc, and Puławska. DNA extraction was performed using Sherlock XP (A&A Biotechnology, Gdańsk, Poland).

### 2.7. Reverse Transcription and Gene Expression

Reverse transcription was performed using the High-Capacity RNA-to-cDNA™ Kit (Applied Biosystems™, Thermo Fisher Scientific, Waltham, MA, USA), following the manufacturer’s protocol. Relative quantification (RQ) of *MALAT1* and adipocyte differentiation marker LPL (lipoprotein lipase) was carried out using a QuantStudio™ 7 Flex Real-Time PCR System (Applied Biosystems™, Thermo Fisher Scientific, Waltham, MA, USA). For the two porcine *MALAT1* isoforms (ENSSSCT00000080860 and ENSSSCT00000077869), primers and probes were designed using Primer Express 3.0 (Applied Biosystems™, Thermo Fisher Scientific, Waltham, MA, USA). The reference genes used for normalisation were RPL27 (Ribosomal Protein L27), RPS29 (Ribosomal Protein L29), and OAZ1 (Ornithine Decarboxylase Antizyme 1) [30]. Gene expression levels were calculated using the ΔΔCt method [31]. The details of all assays, primers and probes are listed in Supplementary Table S1.

### 2.8. MALAT1 Promoter and Exon–Exon Junction Sequencing

DNA and cDNA samples of the ZS and Pietrain breeds were used in this experiment. RNA was isolated from fat-related tissue (subcutaneous dorsal and abdominal adipose tissue, liver and muscle). The reverse transcription procedure is described above. Supplementary Table S1 presents primer pairs, lengths of amplicons employed, type of biological material used as a template (DNA or cDNA), and *MALAT1* isoform tested. PCR products were visualised by agarose gel electrophoresis. Product specificity was additionally assessed by melting curve methods on Quant Studio 7 Flex (Applied Biosystems™, Thermo Fisher Scientific, Waltham, MA, USA)for yielding more than one PCR product, and nested PCR was performed using the same primer pair and 1 µL of the initial PCR product as the template. Fallowing nested PCR, the selected product was excised from the agarose gel and purified using a gel extraction and PCR purification kit (Sigma-Aldrich, Saint Louis, MO, USA). The purified product was sequenced with the Sanger method using the BigDye™ Terminator v3.1 Cycle Sequencing Kit and 3500 Series Genetic Analyzer (Applied Biosystems™, Thermo Fisher Scientific, Waltham, MA, USA). Sequencing chromatograms were analysed using a FinchTV 1.4.0 (Geospiza, Inc. North Tower, WA, USA), and the resulting sequences were aligned using Clustal Omega 1.2 (EMBL-EBI). Promoter analysis covered regions of 1500 pb upstream transcription start site (TSS) for both isoforms. The primer pairs used for promoting amplification are listed in Table S1. All promoter-region amplicons were subjected to Sanger sequencing to identify potential sequence variants within this region.

Differences in putative transcription factor binding sites in *MALAT1* promoter regions were identified using XSTREME, implemented in MEME suite 5.5.8 with the default settings. The identified motifs were matched to transcription factors using TOMTOM, implemented in the MEME suite 5.5.8, a motif comparison tool.

### 2.9. Statistical Analysis

All other statistical analyses were performed using SAS Enterprise Guide 8.3. A two-way ANOVA, with tissue (liver and subcutaneous adipose tissues and muscle) and breed (ZS and Pietrain) as fixed factors, was conducted to analyse gene expression, as well as adipocyte area and diameter with breed (ZS and Pietrain) and anatomical region (dorsal and abdominal) as fixed factors, followed by a post hoc test.

## 3. Results

### 3.1. Histological Structure of Subcutaneous Adipose Tissue

**Figure 1:**
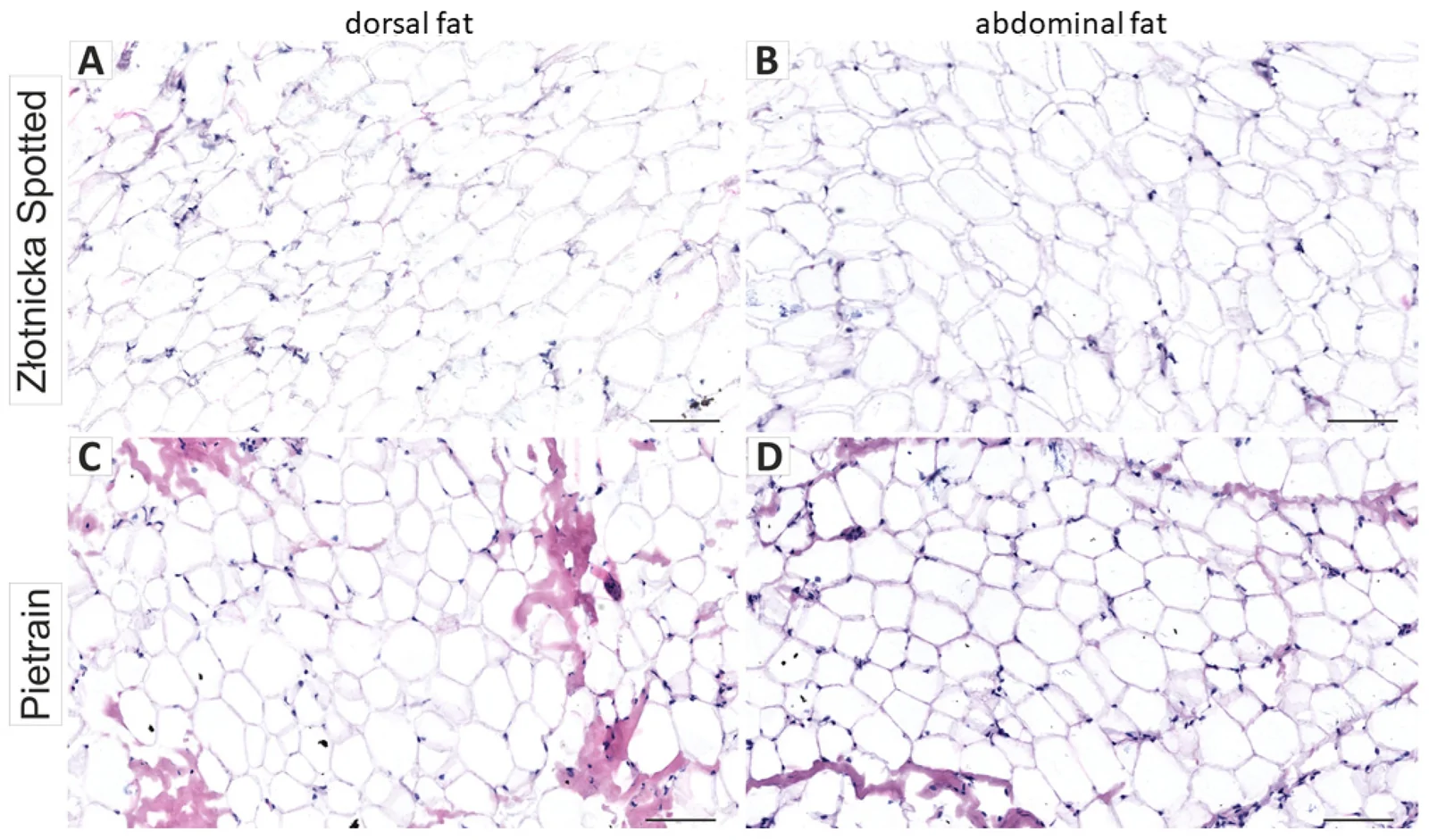
Histological structures of subcutaneous adipose tissue collected from Złotnicka Spotted (ZS) and Pietrain pigs stained with haematoxylin and eosin (H&E). Nuclei are stained in dark purple. Samples were taken from two body regions: (**A**) dorsal fat and (**B**) abdominal fat for ZS and (**C**) dorsal fat and (**D**) abdominal fat for Pietrain pigs. Scale bars: 10 µm.

Adipocytes are visible as large, vacuolated cells with peripherally located nuclei. The adipose tissue of ZS shows larger adipocytes and greater lipid accumulation, whereas the Pietrain pigs display smaller, more compact adipocytes with relatively thicker connective tissue septa. These histological differences correspond to the higher fat-deposition capacity of ZS pigs compared with the lean, muscular phenotype of Pietrain (Figure 1).

Quantitative analysis of adipocyte morphology was performed based on measurements using ImageJ. Adipocytes from ZS pigs were significantly larger than those from Pietrain pigs in both anatomical regions. The largest adipocytes were observed in the dorsal adipose tissue of ZS pigs (mean diameter ≈ 82 µm), while the smallest occurred in the abdominal fat of Pietrain pigs (≈63 µm). These morphometric findings are consistent with the histological observations that support the classification of ZS pigs as representing a high-fat phenotype, whereas Pietrain pigs exhibit a lean phenotype characterised by smaller adipocytes and lower lipid accumulation. Two-way ANOVA was performed separately for adipocyte area and diameter, with breed (ZS and Pietrain) and anatomical region (dorsal and abdominal) as fixed factors. The analysis showed a significant effect of breed on both parameters (*p* < 0.001), indicating that ZS pigs had larger adipocytes than Pietrain pigs. A significant effect of region (*p* < 0.01) was also detected, with adipocytes in the dorsal fat being larger than in the abdominal fat. Additionally, a breed and region interaction was observed (*p* < 0.05), suggesting that regional differences were pronounced in ZS pigs but not in Pietrain pigs (Table 2).

### 3.2. Adipogenic Differentiation and Lipid Accumulation Assay

**Figure 2:**
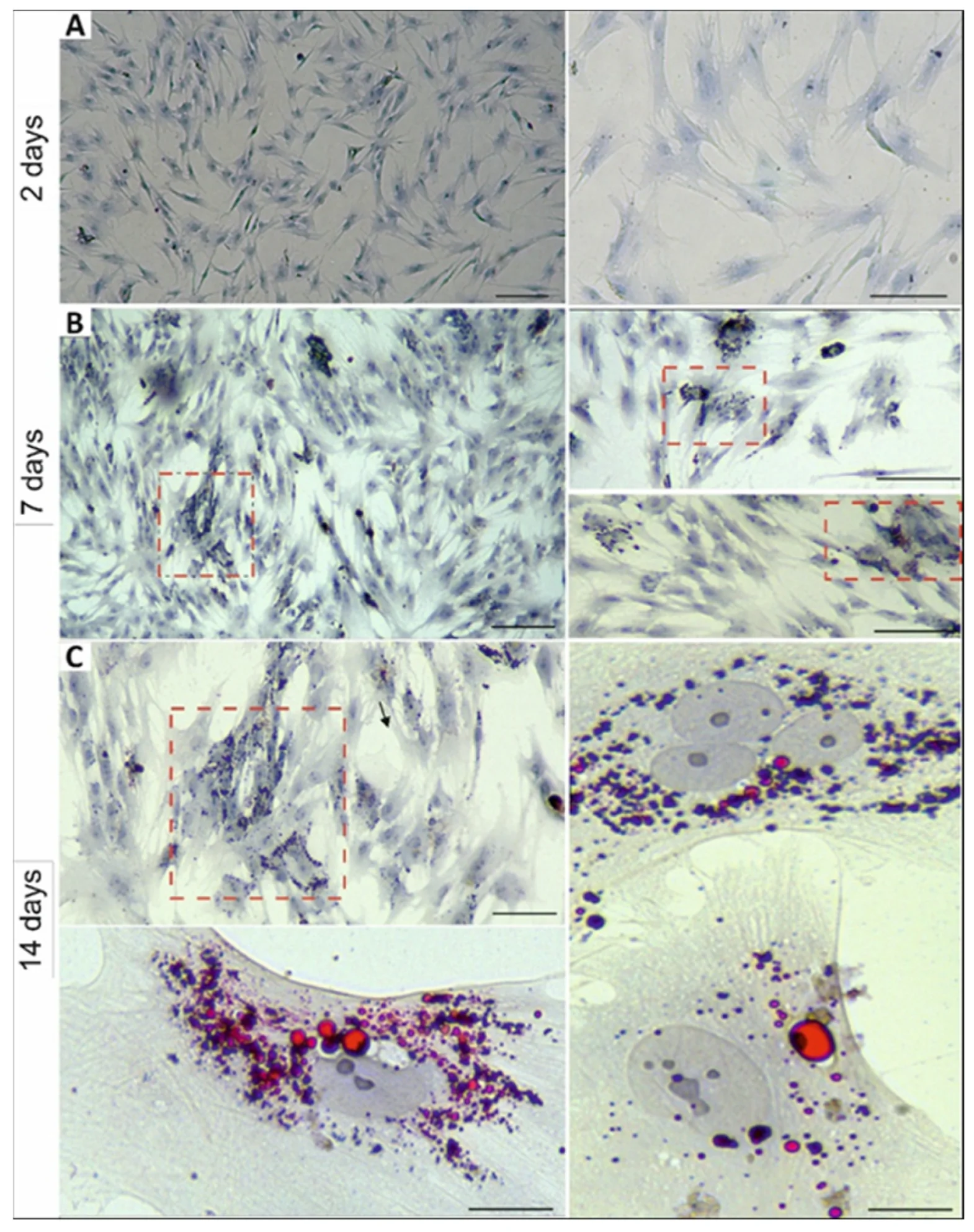
Lipid accumulation during adipogenic differentiation of primary preadipocytes isolated from backfat, visualised using Oil Red O staining (stained red lipid droplets). Progressive adipogenic differentiation was monitored on days 2 (**A**), 7 (**B**), and 14 (**C**), revealing time-dependent accumulation of intracellular lipids (red dashed squares). Oil-Red-O-positive droplets became increasingly prominent, especially at later stages. Insets highlight representative areas of lipid deposition. Scale bars: 10 µm.

In parallel, porcine preadipocytes isolated from the dorsal depot were cultured and subjected to adipogenic differentiation. Morphological changes characteristic of adipogenesis, including increased cell size and lipid droplet formation, were observed over time. After 14 days of differentiation, Oil Red O staining confirmed successful intracellular lipid accumulation (Figure 2).

### 3.3. MALAT1 Promoter Sequencing

**Figure 3:**
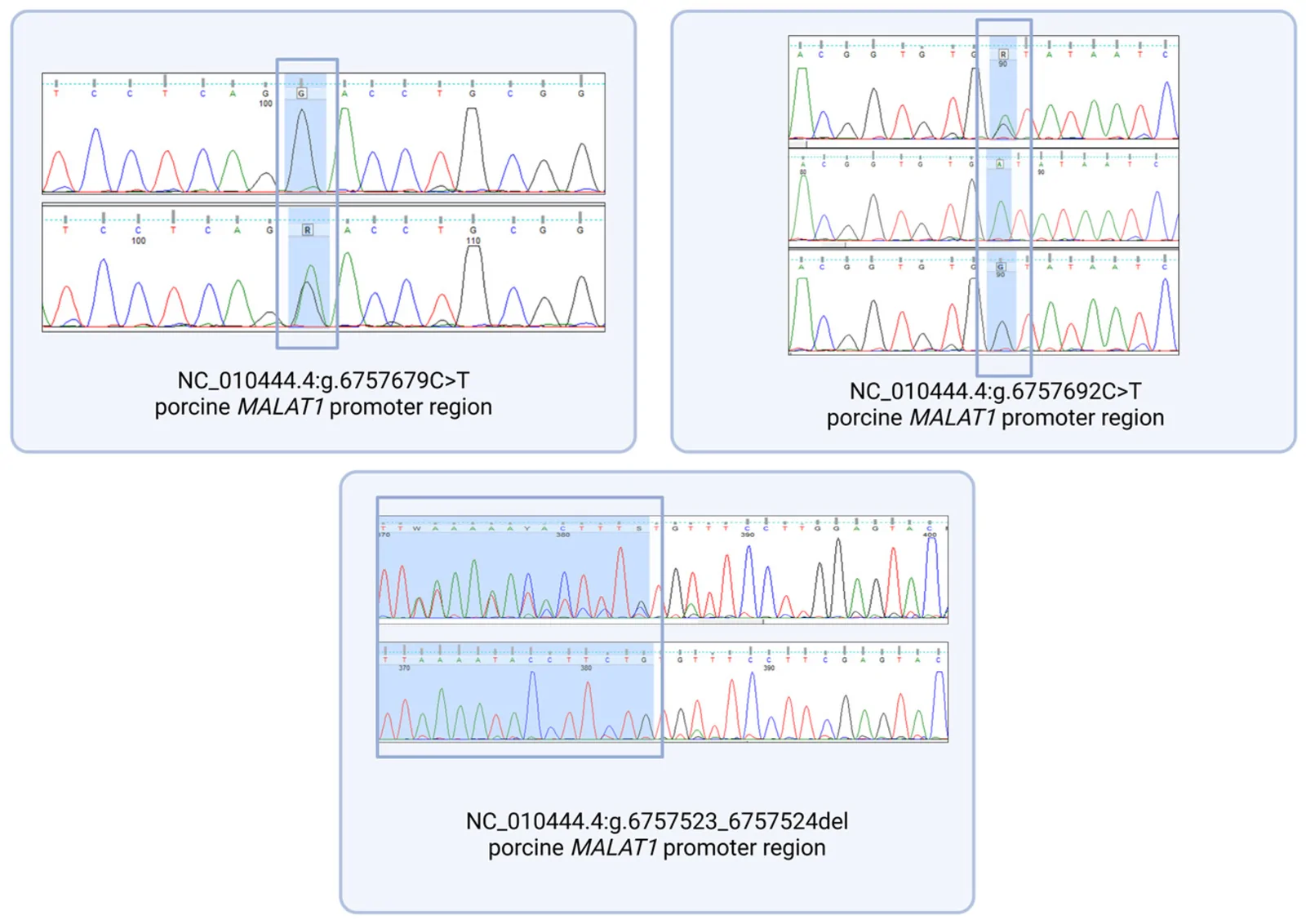
Chromatograms of novel sequence variants identified in the promoter region of porcine *MALAT1*. Blue squares and shadows show new SNPs and deletion identified in the promoter regions of the *MALAT1* gene. Below each chromatogram, the polymorphism name is indicated according to HGVS nomenclature.

Sanger sequencing was carried out for two promoter regions corresponding to the porcine *MALAT1* isoforms ENSSSCT00000080860 and ENSSSCT00000077869. The analysis included seven pig breeds representing both commercial and native Polish populations. In total, 10 polymorphisms were identified across both promoter regions. Seven of these variants had previously been reported (rs338099140, rs329590882, rs321550593, rs333963848, rs318806718, rs319966827, and rs338175674) (Ensembl genome browser 115). In addition, the following three novel variants were identified in the present study: NC_010444.4:g.6757523_6757524del, NC_010444.4:g.6757692C>T, and NC_010444.4:g.6757679C>T (Figure 3). All identified porcine *MALAT1* promotor variants were submitted as haplotypes to the NCBI database under accession numbers OR941310–OR941322.

The allele frequency of these polymorphisms was estimated in seven breeds (Table 3). For example, the minor allele of rs338099140 occurred at a frequency of 27% in ZW but was rare in remaining breeds. The rs329590882 variant was found at a low frequency in PL. The rs321550593G allele showed the highest frequency (18.8%) in Złotnicka Spotted, while rs695444210(-) exceeded 30% in ZW. The newly identified deletion g.6757523_6757524del was observed exclusively in Duroc and ZS pigs. Common variants such as rs333963848G, rs318806718G, and rs338175674G were detected across all breeds, although at varying frequencies. Notably, the novel SNP NC_010444.4:g.6757718G>A exhibited the highest frequency (31.3%) in Pietrain pigs.

### 3.4. In Silico Prediction of Transcription Factor Binding in MALAT1 Promoters

All polymorphisms identified within the promoter regions of the porcine *MALAT1* gene were analysed for their potential effects on transcription factor binding sites (TFBSs) using the XSTREME module in MEME Suite. Multiple sequence motifs corresponding to putative TFBSs were predicted (Table S2). Based on our previous transcriptomic analyses of liver and backfat tissue stratified according to fat-deposition levels [18,32] (GEO accession GSE160436), the expression of selected transcription factors was confirmed in the relevant tissues. Particular attention was given to TFs predicted to bind at polymorphism sites within the *MALAT1* promoter regions. The rs329590882 variant, which was rare in Duroc and PL, was predicted to affect binding sites for NFAT5 (Nuclear Factor of Activated T-Cells 5), ZNF75D (Zinc Finger Protein 75D), and ERF (ETS2 Repressor Factor). The rs321550593 variant, which was relatively frequent in ZS, was associated with predicted binding motifs for SOX13 (SRY-Box Transcription Factor 13), SRF (Serum Response Factor), and HOMEZ (Homeodomain Leucine Zipper-Containing Factor). The novel NC_010444.4:g.6757692C>T variant, which was frequent in Pietrain, was located within predicted binding sites for MSX1 (Msh Homeobox 1) and FOXK1 (Forkhead Box K1). In addition, rs318806718, which was rare in Duroc, was located within the predicted TFBSs for PBX3 (Pre-B-Cell Leukaemia Homeobox 3), NR1D1 (Nuclear Receptor Subfamily 1 Group D Member 1), and XBP1 (X-Box Binding Protein 1). The rs319966827 variant, which was rare in the Złotnicka breeds, overlapped with predicted binding motifs for BCL6B (B-Cell CLL/Lymphoma 6 Member B) and NFATC1 (Nuclear Factor of Activated T-Cells 1). These findings suggest that sequence variation within the *MALAT1* promoter may influence regulatory interactions in a tissue-specific and breed-dependent manner (Table S2).

### 3.5. MALAT1 Gene Expression Analysis

**Figure 4:**
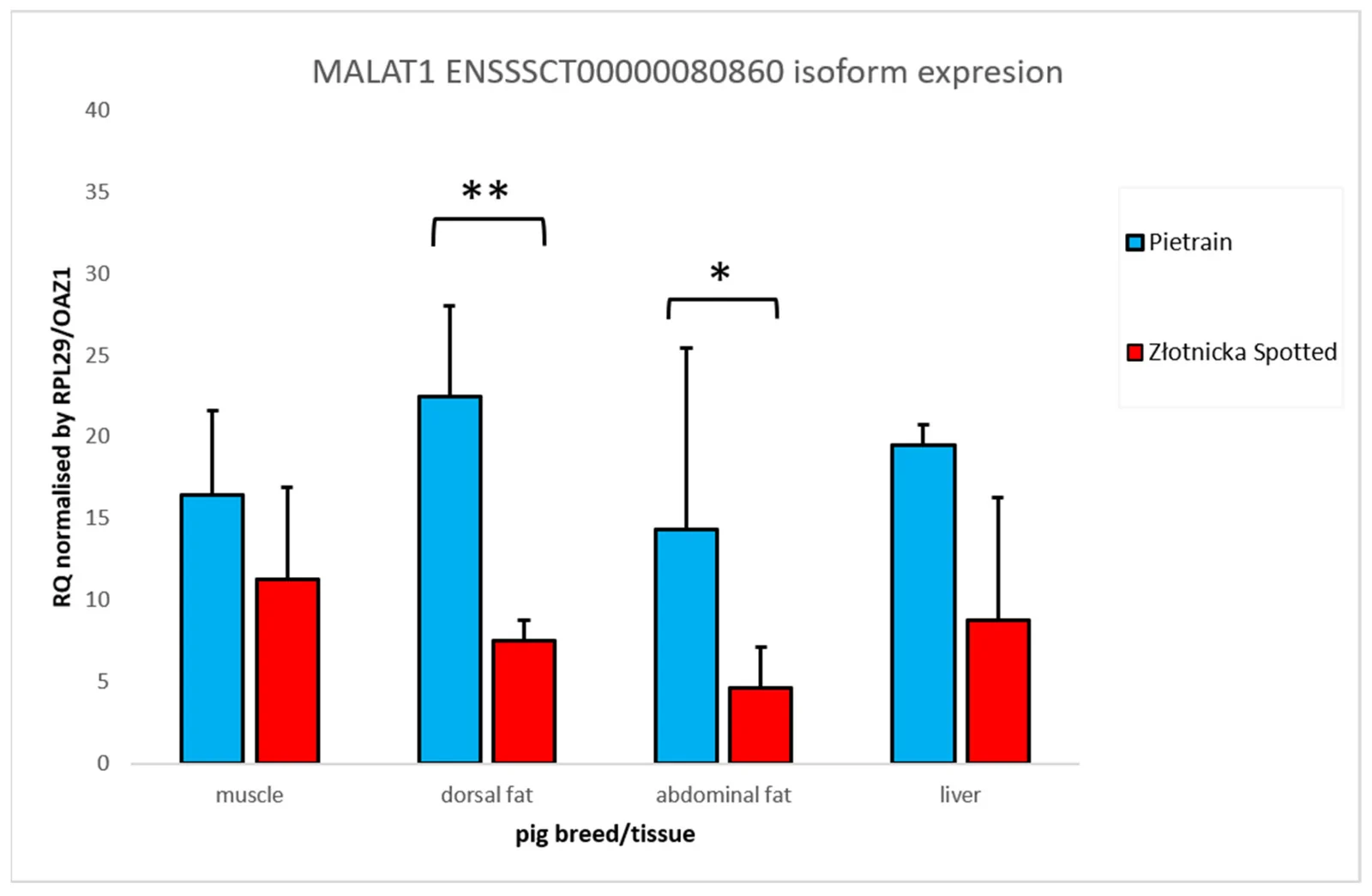
lncRNA *MALAT1* expression patterns in different tissue related to fat deposition in two pig breeds: ZS, representing a fatty phenotype, and Pietrain, representing a lean phenotype.* *p*-value < 0.05., ** *p*-value < 0.01.

The expression profile of *MALAT1* was assessed both during adipogenic differentiation and in porcine tissues associated with fat deposition. Isoform-specific quantification detected only the ENSSSCT00000080860 isoform. Its expression was significantly higher in the subcutaneous adipose tissues of Pietrain pigs, with an approximately three-fold increase in both backfat and abdominal fat compared with ZS pigs (Figure 4). No amplification signal was detected using the primer–probe set specific to the ENSSSCT00000077869 isoform, which was consistent with our subsequent observations concerning this transcript.

**Figure 5:**
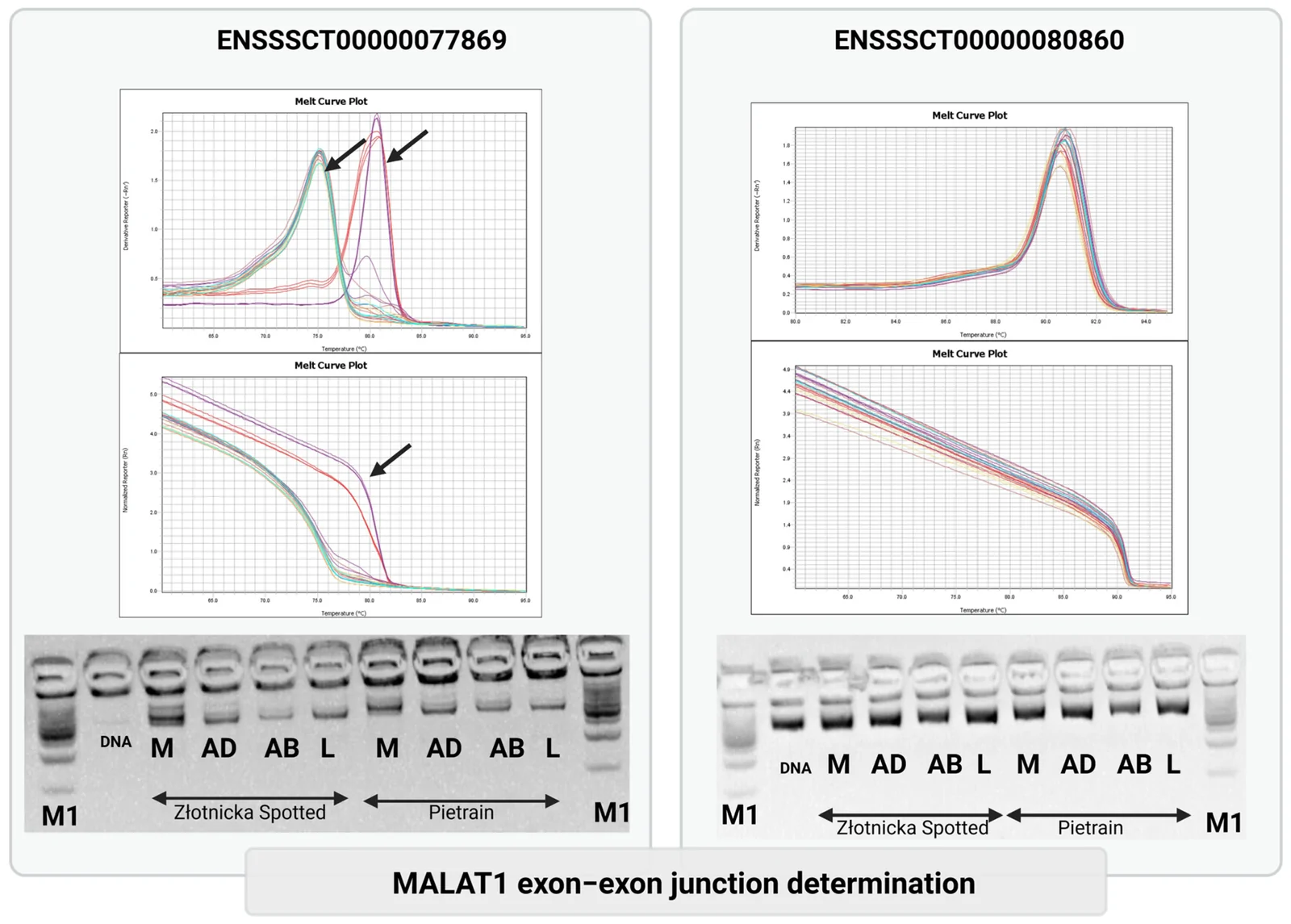
Melting plots and gel band visualisation for ENSSSCT00000077869 and ENSSSCT00000080860 products. The analysis included Złotnicka and Pietrain pigs and four types of tissue (M—muscle, AD—dorsal adipose, AB—backfat adipose and L—liver tissue). M1—marker. Arrows show differences between analysed samples. Melting curves for individual analysed samples are shown in different colors.

**Figure 6:**
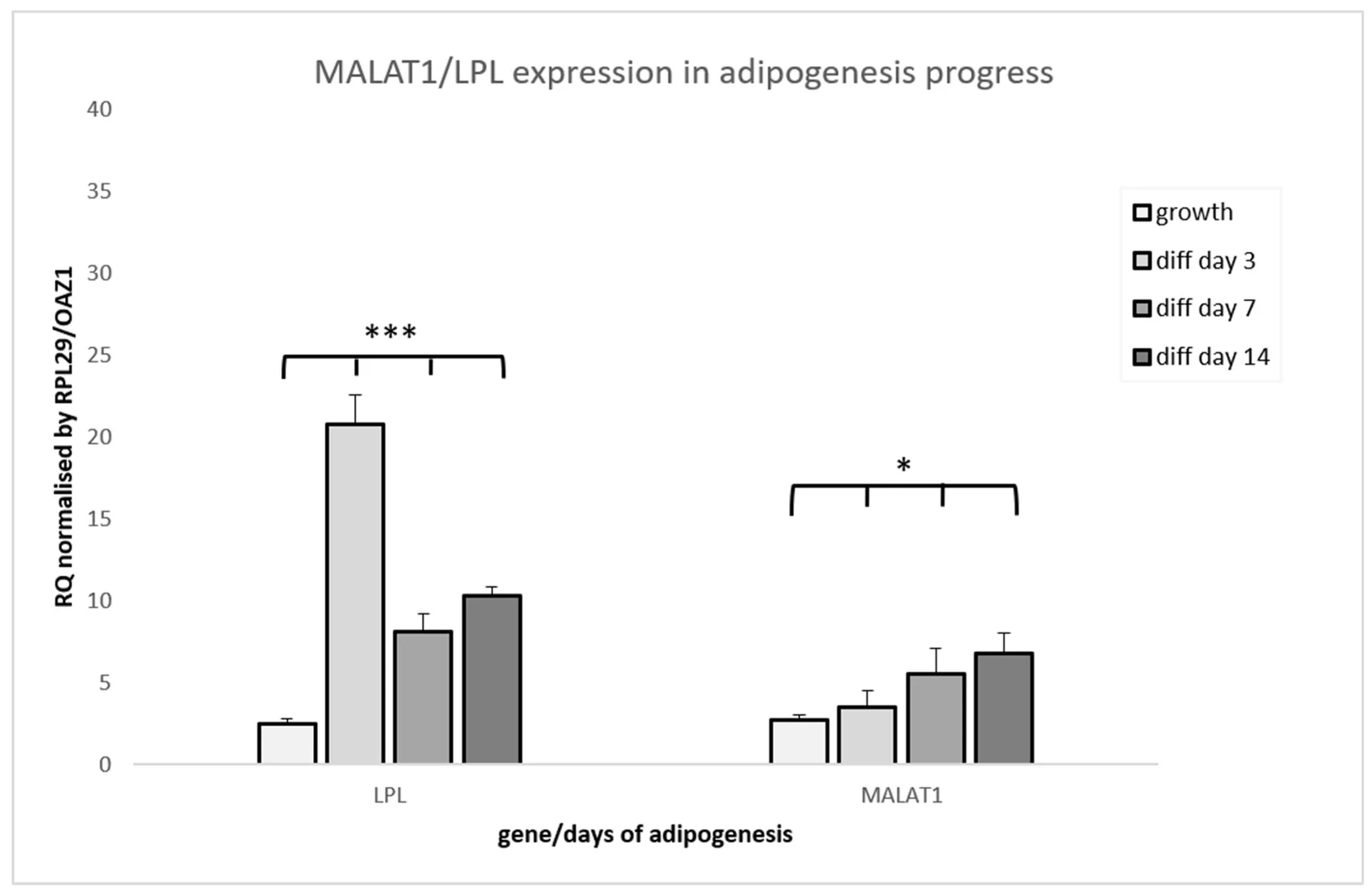
Expression pattern of lncRNA *MALAT1* during adipogenesis in primary preadipocytes isolated from backfat tissue of Złotnicka Spotted pigs. Expression levels were monitored from days 0 to 14 of differentiation. “diff” indicates the day of differentiation. Expression of the adipogenic marker LPL was used to confirm adipocyte maturation. * *p*-value < 0.05, *** *p*-value < 0.001.

In contrast, amplification of regions shared by both isoforms, yielded two distinct PCR products. Melting curve analysis revealed reproducible differences in melting temperatures among tissues, suggesting tissue-specific variation in transcript composition (Figure 5). Agarose gel electrophoresis confirmed the presence of distinct amplicons. Subsequent nested PCR and Sanger sequencing identified 20 exon–exon junction variants. These included a transcript corresponding to ENSSSCT00000077869 and a variant of ENSSSCT00000080860 containing a retained intronic fragment, consistent with our previous observations [19]. These findings indicate the presence of additional porcine *MALAT1* transcript variants that have not yet been annotated in the current genome databases. During the in vitro adipogenic differentiation of primary preadipocytes, *MALAT1* expression progressively increased from day 0 to day 14, in parallel with adipocyte maturation. This expression pattern was consistent with the upregulation of LPL, a well-established marker of adipogenic differentiation (Figure 6). It was also supported by morphological changes and intracellular lipid accumulation observed via Oil Red O staining (Figure 2).

## 4. Discussion

*MALAT1* belongs to the class of lncRNAs, whose biological functions have not yet been fully elucidated. Initially, lncRNAs were believed to primarily be antisense molecules involved in transcripts’ inactivation [33]. Subsequent studies have demonstrated their diverse roles in the regulation of gene expression. As described in the Introduction, *MALAT1* has a well-established role in carcinogenesis and metastasis. Nevertheless, an increasing body of evidence also implicates *MALAT1* in molecular processes associated with fat deposition and obesity. Yan et al. [34] proposed that *MALAT1* promotes hepatic fat accumulation by increasing the stability of the SREBP-1c protein. Ebrahimi et al. [35] analysed *MALAT1* expression in subcutaneous adipose tissue (SAT) and found no significant differences between women with and without obesity. In contrast, a study published in 2024, involving more than 300 women classified as overweight and obese, investigated the correlation between *MALAT1* expression and the Cholesterol–Saturated Fat Index [15]. The authors reported a significant positive inter-action between *MALAT1* expression and this index in relation to visceral adiposity and body adiposity indexes, indicating that further functional studies are needed to clarify this concept. Moreover, *MALAT1* expression was positively correlated with the expression of sterol regulatory element-binding protein 1c (SREBP-1c) and peroxisome proliferator-activated receptor gamma (PPAR-γ), as well as with that of their downstream targets, fatty acid synthase (FASN) and acetyl-CoA carboxylase alpha (ACACA). A recent study investigating the effects of *MALAT1* on steatosis in HepG2 cells showed that *MALAT1* silencing significantly altered the expression of *FASN*, *ACADL*, *CPT1A*, and *MTTP* [36].

In the present study, we investigated the relationship between *MALAT1* expression, adipogenesis and fat deposition in pigs. In the first experiment, *MALAT1* expression was profiled during adipogenesis in primary porcine preadipocytes derived from subcutaneous adipose tissue and in porcine tissues associated with carcass fatness. *MALAT1* expression increased significantly over the course of adipogenic differentiation, suggesting its involvement in this process. Tissue-specific analysis showed that expression of the *MALAT1* isoform ENSSSCT00000080860 was higher in the SF of Pietrain pigs, which have a leaner body composition than ZS pigs. Kociucka et al. [37] reported that Pietrain pigs had considerably smaller adipocytes in subcutaneous adipose tissue than PL pigs, with mean diameters of approximately 55 and 70 µm, respectively. The corresponding backfat thickness values were 0.965 and 1.683. However, Pietrain pigs had a greater number of adipocytes per cm2. In the present study, we report a mean backfat thickness of 3.08 cm in ZS and 1.14 cm in Pietrain pigs. Our histological analysis also showed that Pietrain pigs had smaller, more compact adipocytes. In dorsal adipose tissue, their mean adipocyte area and diameter were 3428.4 µm^2^ and 66.1 µm compared with 5297.8 µm^2^ and 82.1 µm in ZS pigs (*p*-value < 0.0001). The higher *MALAT1* expression observed in leaner Pietrain pigs, despite their smaller adipocyte size, suggests that *MALAT1* may be more closely associated with preadipocyte proliferation and differentiation than with adipocyte hypertrophy. However, this hypothesis requires direct functional validation. Caron et al. [38] showed that *MALAT1* silencing reduced adipocyte differentiation capacity. Moreover, in their study, shear stress was correlated with the downregulation of *MALAT1* expression in adipocytes derived from mesenchymal stem cells [39]. In our experiments, the progressive increase in MALAT1 expression during adipogenesis was consistent with these findings. Similar results were reported by Han et al. [39], who revealed that *MALAT1* knockdown significantly inhibited adipogenesis. This effect may be related to the ability of *MALAT1* to regulate PPAR-γ expression, a key transcription regulator of numerous adipogenic genes. *MALAT1* has also been identified as the most abundant lncRNA in exosomes from human adipose-tissue-derived stem cells (hADSCs) [40]. Han et al. [39] suggested that *MALAT1* promotes adipose tissue formation. However, in the present study, *MALAT1* expression was significantly higher in both subcutaneous tissues of Pietrain pigs than in the fatty ZS pigs. By contrast, Ebrahimi et al. [35] found lower *MALAT1* expression in obese mice than in control animals. *MALAT1* knockdown had no stimulatory or inhibitory effect on diet-induced fat accumulation or lipid homeostasis. Our findings were unexpected because, in our previous study, higher *MALAT1* was correlated with greater fatness within a population of ZW pigs. In summary, these observations suggest that *MALAT1* in adipose tissue may depend on the stage of adipogenesis, tissue context, or genetic background, which supports our above hypothesis. Our additional observation of the *MALAT1* transcript structure also newly identified that exon–exon junctions differed among tissue types, suggesting the presence of tissue-specific *MALAT1* transcript variants that may perform distinct biological functions [41]. In addition, we analysed the promoter regions of two porcine *MALAT1* isoforms and identified substantial variation among pig breeds differing in adipose and muscle tissue contents. The rs329590882 variant was predicted to influence the binding site for transcription factors NFAT5 and ZNF75D within the porcine *MALAT1* promoter region. NFAT5 has been implicated in the regulation of WAT to BAT conversion. In mice fed a high-fat diet, NFAT5 deficiency led to the formation of small, focal lipid-rich lesions in the aorta [42]; promoted cholesterol accumulation in vascular smooth muscle cells; and reduced the expression of genes involved in the management of oxidative stress or lipid handling, such as *SOD1*, *PLIN2*, *FABP3*, and *PPARD*. Thus, NFAT5 may affect lipid accumulation through several mechanisms. Although it may contribute to obesity by inhibiting the conversion of white fat tissue into “beige” or brown-like adipocytes, it also participates in cellular stress responses and inflammatory response, which may indirectly influence lipid processing and storage [43]. In the present study, the frequency of rs329590882 genotypes differed by 44% between Pietrain and ZW. The A allele, which was predicted to favour NFAT5 binding within the *MALAT1* promoter region, predominated in Pietrain pigs, reaching a frequency of 58.3%. Pietrain pigs showed three-fold higher *MALAT1* expression than ZS pigs. This observation suggest a potential association between rs329590882, NFAT5 binding, and *MALAT1* expression; however, this relationship needs confirmation using a functional promoter assay. ZNF75D belongs to the Zinc Finger Protein family and has been suggested to participate in adipogenesis and fat accumulation. Nevertheless, its influence on the expression of key adipogenic genes and its precise role in adipose tissue biology remain to be elucidated.

The rs321550593 variant, located upstream of *MALAT1*, showed the highest frequency in ZS (18.8%), in which a lower *MALAT1* expression was observed in both subcutaneous tissues (abdominal and dorsal tissue) was observed. Our in silico analysis predicted that this polymorphism may affect binding sites for several TFs, including SOX13 and SRF. SOX13 has been reported to promote the differentiation of mesenchymal stem cells into brown adipocytes through interactions with other adipogenic TFs, including SIX1 and RREB1 [44]. Moreover, SOX13 knockdown impairs this process and overexpression enhances it. SOX13’s role during preadipocyte proliferation is not exactly explained, though this TF is rather pinpointed as a repressor of proliferation and has been broadly described in cancer cells [44,45]. In our study, an increased frequency of the rs321550593 alternate variant favouring SOX13 binding in ZS was observed, and thus, this TF could be involved in the regulation of *MALAT1*; however, this should be investigated in a future study. In turn, SRF is a known inhibitor of preadipocyte proliferation and BAT adipogenesis, particularly during the early stages of commitment and differentiation, through negative regulation of the TGF-β pathway [46]; suppression of SRF-MRTF leads to enhanced adipocyte development. Nevertheless, in our study, an increased frequency of rs321550593 with the G allele in ZS pigs did not favour SRF binding; thus, SRF could be associated with the increased *MALAT1* expression observed in Pietrain pigs.

The rs318806718 *MALAT1* promoter variant seems to affect PBX3 and XBP1, both of which are crucial factors determining fat deposition; PBX3 is involved in preadipocyte proliferation because it influences cell cycle progression and activates the AKT signalling pathway [47]. XBP1 is a key factor in adipogenic differentiation that suppresses Wnt10b and decreases the β-catenin signalling pathway [48,49]. On the other hand, XBP1 promotes hypertrophy by activating adipogenesis in preadipocytes, which is related to the regulation of key adipogenic transcription factors like a C/EBPα. In our study, we observed a 30% increase in the frequency of the rs318806718G allele, promoting binding of PBX3 and repressing XBP1 in Pietrain pigs (58.3% of rs318806718G). Due to the more numerous and non-hypertrophic adipocytes observed in Pietrain pigs, as well as the three-fold increase in *MALAT1* expression, we conclude that PBX3 could be involved in *MALAT1* expression and, further, the preadipocyte proliferation process. In turn, the preference for XBP1 binding in ZS pigs could be associated with preadipocyte differentiation and, further, hypertrophy.

## 5. Conclusions

The function of lncRNAs is still not fully understood. Although we can preliminarily assign distinct biological functions to these molecules, it is often unclear at which molecular level each lncRNA can regulate the expression of other genes. *MALAT1* has been extensively studied in humans; however, in farm animals, particularly pigs, the available knowledge is still very limited and mainly restricted to its chromosomal localisation. There is currently a significant lack of information regarding the biological function and regulatory role of lncRNAs in pigs, making studies in this field highly relevant and necessary. Our initial observations revealed that *MALAT1* may be associated with adipogenesis and fat deposition in pigs, providing novel insights into the potential role of this lncRNA in porcine lipid metabolism. Although these findings are preliminary, they contribute important new knowledge to the poorly explored area of lncRNA function in swine. Nevertheless, the relatively small sample size should be considered when interpreting the findings, and future studies should include larger animal populations. Functional experiments involving *MALAT1* silencing and overexpression in primary porcine preadipocytes will also be required to clarify the molecular mechanisms underlying *MALAT1*-mediated regulation.

## Figures and Tables

**Table 1:** Characteristics of Złotnicka Spotted (ZS) and Pietrain pigs representing high and low fat-deposition traits, mean ± SD.

Traits	ZS (*n* = 4)			Pietrain (*n* = 4)				
	Mean		SD	Mean		SD	*p*-Value	Group Diff. *
Daily gain (g)	639	A	18.7	1031	B	108	0.00353	38%
Yield percentage	75.2	A	0.49	77.5	B	0.62	0.0013	3%
Peritoneal fat (kg)	0.80		0.31	0.42		0.13	0.06	36%
Tenderloins (kg)	0.30	A	0.06	0.46	B	0.05	0.008	48%
Ham mass (kg)	8.82	A	0.69	11.7	B	0.78	0.00172	25%
Backfat thickness at the K1 point (cm)	3.38	A	0.75	0.88	B	0.15	0.004495	74%
Ham fat mass with skin (kg)	2.46	A	0.24	1.15	B	0.23	0.00025	53%
Loin fat mass with skin (kg)	2.45	A	0.49	0.83	B	0.21	0.003	66%
Bacon with ribs (kg)	6.2	A	0.13	5.1	B	0.16	6.39 × 10^−5^	18%
Fat over shoulder thickness (cm)	3.6	A	0.5	1.9	B	0.19	0.00301	56%
Lumbar fat I thickness (cm)	3.35	A	0.65	1.00	B	0.31	0.001951	69%
Lumbar fat II thickness (cm)	2.98	A	0.60	0.7	B	0.0	0.0036	77%
Lumbar fat III thickness (cm)	3.39	A	0.68	0.85	B	0.11	0.00337	75%
Average backfat thickness (cm)	3.08	A	0.50	1.14	B	0.15	0.00214	63%
Loin eye area (cm^2^)	32.05	A	4.95	66.8	B	1.53	0.000294	52%

SD—standard deviation. * Group diff.—the difference between groups in percentage. Values with the same letters belong to the same statistical group (A, B = *p* < 0.01). K1—backfat thickness at the K1 point (cm), describing backfat thickness over the lateral edge of the longissimus dorsi muscle.

**Table 2:** Morphometric characteristics of adipocytes in subcutaneous adipose tissue of Złotnicka Spotted (ZS) and Pietrain pigs (H&E staining).

Breed	Region	Mean Area (µm^2^)	SD Area (µm^2^)	Mean Diameter (µm)	SD Diameter (µm)	*n*
ZS	back fat	3995.5 Aa	680.4	71.3 Aa	6.1	50
	dorsal fat	5297.8 Cb	1392.7	82.1 Cb	10.8	50
Pietrain	back fat	3063.8 Bc	847.9	62.6 Bc	8.6	50
	dorsal fat	3428.4 Dd	1452.6	66.1 Dd	14.0	50

A, B, C, D—capital letters show significant differences among groups at a *p*-value < 0.0001; a, b, c d—lower letters show significant differences among groups at a *p*-value < 0.05.

**Table 3:** The frequencies of indicated minor alleles of porcine *MALAT1* mutations for promoter regions accompanied both *MALAT1* isoforms ENSSSCT00000080860 and ENSSSCT00000077869, including 7 pig breeds representing commercial and native Polish pigs.

Pig Breed	Porcine *MALAT1* Promoter Variations									
	rs338099140 (C)	rs329590882 (A)	rs321550593 (C)	rs695444210 (-)	New (-)	New (C)	New (T)	rs318806718 (C)	rs319966827 (C)	rs338175674 (C)
Puławska	0	68.8	2.1	0	0	10.4	8.3	81.3	18.8	18.8
Duroc	0	6.3	0	0	8.3	29.2	2.1	4.2	46.7	46.7
Polish Large White	2.1	60.4	0	2.1	0	4.2	0	62.5	18.8	18.8
Pietrain	0	58.3	6.3	0	0	4.2	21	58.3	37.5	35.4
Polish Landrace	4.2	2.1	2.1	4.2	0	23	0	52.1	18.8	22.9
Złotnicka Spotted	0	14.6	18.8	0	4.2	8.3	0	39.6	8.3	8.3
Złotnicka White	27.1	16.7	8.3	31.3	0	31.3	0	33.3	0	31.3
Total (%)	4.8	32.4	5.4	5.4	1.8	15.8	4.5	46.7	19	23.8

## Data Availability

The sequence data obtained after Sanger sequencing analysis were submitted to the GenBank NCBI (accession no. OR941310-OR941322).

## Supplementary Materials

The following supporting information can be downloaded at: https://www.mdpi.com/article/10.3390/genes17080888/s1.

## Abbreviations

The following abbreviations are used in this manuscript:

AB	Backfat adipose tissue
AD	Dorsal subcutaneous adipose tissue
BAT	Brown adipose tissue
BCL6B	B-Cell CLL/Lymphoma 6 Member B
ceRNA	Competing endogenous RNA
FASN	Fatty acid synthase
H&E	Haematoxylin and eosin
*MALAT1*	Metastasis-Associated Lung Adenocarcinoma Transcript 1
L	Liver
lncRNA	long non-coding RNA
M	Muscle
miRNA	MicroRNA
ncRNA	Non-coding RNA
NFAT5	Nuclear Factor of Activated T-Cells 5
NFATC1	Nuclear Factor of Activated T-Cells 1
PBX3	Pre-B-Cell Leukaemia Homeobox 3
PPAR-γ	Peroxisome proliferator-activated receptor gamma
PLW	Polish Large White
PL	Polish Landrace
SAT	Subcutaneous adipose tissue
SF	Subcutaneous fat
SOX13	SRY-Box Transcription Factor 13
SIRT1	Sirtuin 1
SRA	Steroid receptor RNA activator
SREBP-1c	Sterol Regulatory Element-Binding Protein 1c
SRF	Serum response factor
TFBS	Transcription binding site
WAT	White adipose tissue
XBP1	X-Box Binding Protein 1
ZNF75D	Zinc Finger Protein 75D
ZS	Złotnicka Spotted
ZW	Złotnicka White
