# Supplementary material for: Tracing of Porcine lncRNA MALAT1 Shaped by Tissue Localisation and Adipogenesis
Source: Genes (Basel). 2026 Jul 29;17(8):888. doi: 10.3390/genes17080888 (PMC13512537; doi:10.3390/genes17080888)

Table S1. Primers and probes used in qPCR analysis

Gene		Primers and Probe<br>or<br>Taqman Gene Expression Assay ID	Amp. length [bp]	Matrix	Label	Reference sequences accesion numbers	Exon Boundary
			Gene expression				
RPL27	60S ribosomal protein L27<br>**housekeeping gene**	5`NED – CGGTCATCGTAAAGAA<br>CGCTACTCCGGACGCAAA<br>GGTCTGAGGTGCCATCATCA	55	cDNA	NED	ENSSSCG00000025507	1-2
OAZ1	ornithine decarboxylase antizyme 1<br>**housekeeping gene**	Ss03387505_u1	177	cDNA	FAM	NM_001122994.1	5-5
RPS29	Ribosomal protein S29<br>**housekeeping gene**	Ss03391548_g1	71	cDNA	VIC	NM_001001633.2	2-3
MALAT1- isoform1	Metastasis Associated Lung Adenocarcinoma Transcript 1 Isoform 1	5`FAM – GATAGAGCGTCCTAGAGCGG<br>CGGGAGCCATGCGATGTG<br>AAACAAAGCAGAACCCACGA	60	cDNA	FAM	ENSSSCT00000080860	1-2
MALAT1- isoform1	Metastasis Associated Lung Adenocarcinoma Transcript 1 Isoform 1	F- CGGGAGCCATGCGATGTG<br>R-AAACAAAGCAGAACCCACGA	200	cDNA	Evagreen	ENSSSCT00000080860	1-2
MALAT1- isoform2	Metastasis Associated Lung Adenocarcinoma Transcript 1 Isoform 2	5`VIC - AAGAGTTGCTTCATATCAT<br>TCATGGTCTGCGTTTCTCATTATC<br>GCAATACTGCAGATGCAAGTTAAACT	61	cDNA	VIC	ENSSSCT00000077869	1-2
MALAT1- isoform2	Metastasis Associated Lung Adenocarcinoma Transcript 1 Isoform 2	F-TTCAGAGCAAAGGAAGTGGC<br>R-TGAGTAAGGGACCTAGGACC	298	cDNA	Evagreen	ENSSSCT00000077869	1-2
LPL	lipoprotein lipase	Ss03394608_m1	78	cDNA	FAM	NM_214286.1	5-6

			Sanger sequencing			
MALAT1- promoter1	Metastasis Associated Lung Adenocarcinoma Transcript 1 Isoform 1	F1-ATTTTCCTCCCAGCTCCACT<br>R1- TGCTTTGGAGGCTAGGAAAG<br>F2-AGTGGTAAAGAGTGGCTGTCA<br>R2- AAGCGTTTCCTGCCTCTCA	772<br>684	DNA	ENSSSCT00000080860	Promoter1
MALAT1 promoter 2	Metastasis Associated Lung Adenocarcinoma Transcript 1 Isoform 2	F1- GGGGTGGTCTTAACAGGGAA<br>R1-TCCAAAAGAGAACCACACACT<br>F2-TATTTTGGGGTG GTCTTAACAGG<br>R2-CCCCAATCCAACACTTTCCT	691<br>600	DNA	ENSSSCT00000077869	Promoter2
MALAT1- isoform 1 Exon-exon	Metastasis Associated Lung Adenocarcinoma Transcript 1 Isoform 1	F-AAAATCGGCCTACATCCCCA<br>R-CTCGAAGCTGTAGAGGGAAGT	695<br>786	cDNA,<br>DNA	ENSSSCT00000080860	1-2
MALAT1- isoform 2 Exon-exon	Metastasis Associated Lung Adenocarcinoma Transcript 1 Isoform 2	TGCCTCGAGTGAAGTGAGTT<br>GGGCTTGGGAAAGGAAAGTG	579<br>1 216	cDNA,<br>DNA	ENSSSCT00000077869	1-2

Amp.- amplicon

Table S2. Sequence motifs identified within MALAT1 promoter regions corresponding to putative TFBSs. Analysis has been performed using MEME suite 5.5.8 XSTREME - Motif Discovery and Enrichment Analysis (default setting). Motifs were matched to transcription factors using TOMTOM (Motif comparison tool).

<table>
<tr><th>MALAT1 promoter mutation</th><th>Transcription factor</th></tr>
<tr><td>rs338099140</td><td>
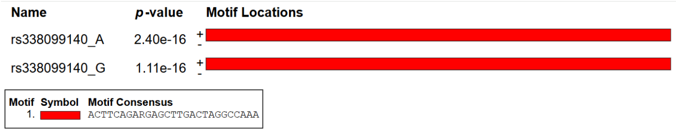

ESSRA
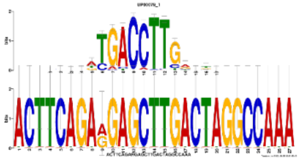
PAX2
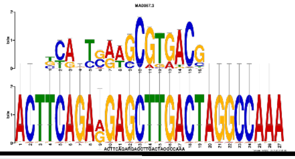
</td></tr>
<tr><td>rs329590882</td><td>
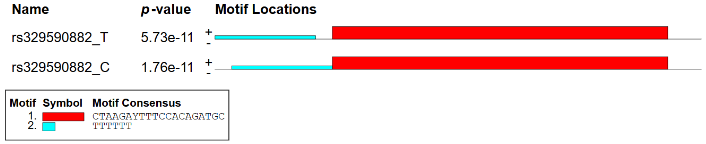

ERF::NHLH1
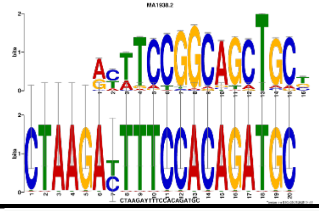
NFAT5
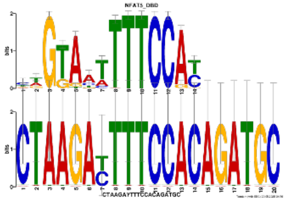
ZNF75D
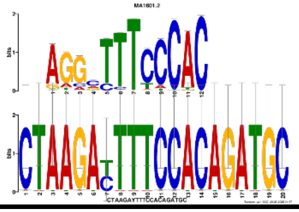
</td></tr>
<tr><td>rs321550593</td><td>
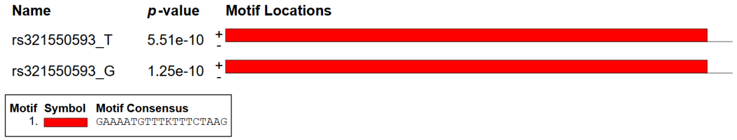

</td></tr>
</table>

SOX13

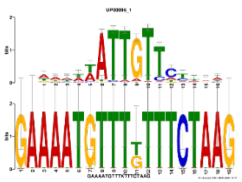

SRF

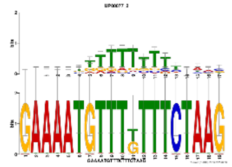

FOXJ1

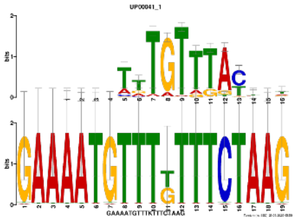

FOXD2

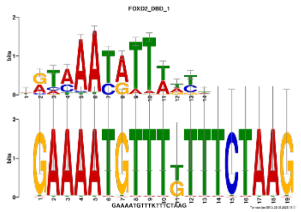

FOXL1

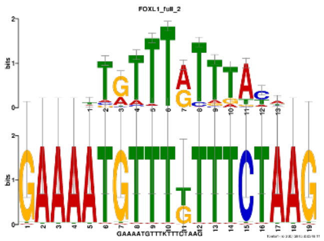

HOMEZ

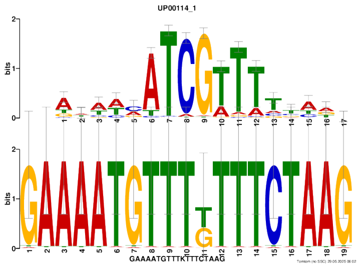

FOXC2

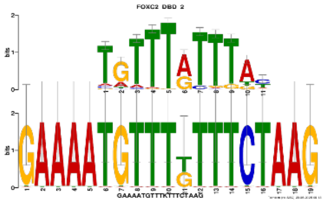

rs695444210

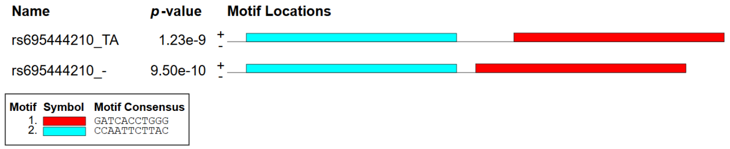

CCAATTCTTACA[TA/-]GATCACCTGGG

NC_010444.4:g.6757523_6757524del

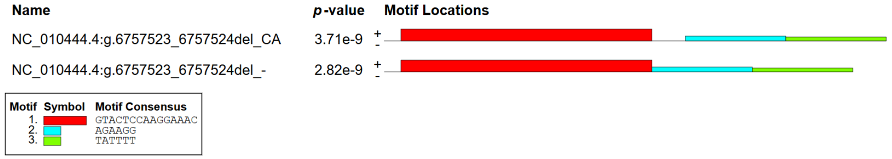

GTACTCCAAGGAAA[CA/-]CAGAAGGTATTTT

NC_010444.4:g.6757692C>T

Name	*p*-value	Motif Locations
NC_010444.4:g.6757705A>G_A	1.85e-17	
NC_010444.4:g.6757705A>G_G	7.98e-18	

Motif	Symbol	Motif Consensus
1.		CACGAATAAAATGGCRATCAAGACGGTGT

YY2

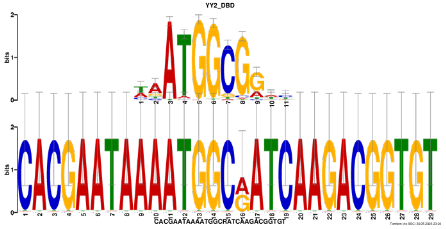

MSX1

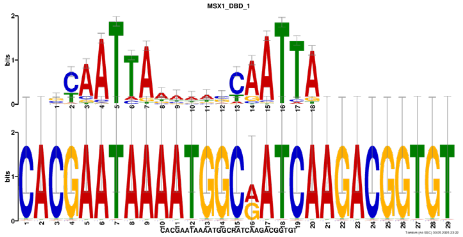

HESX1

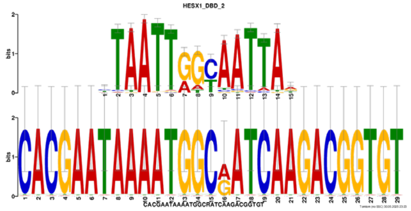

FOXA2

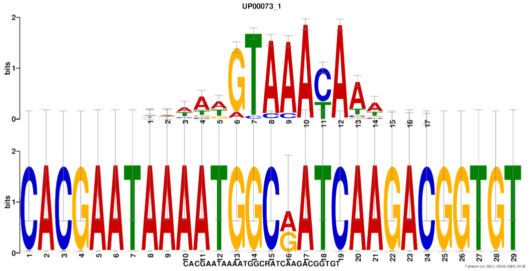

MSX3

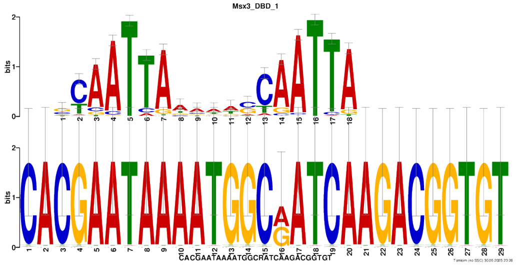

NANOG

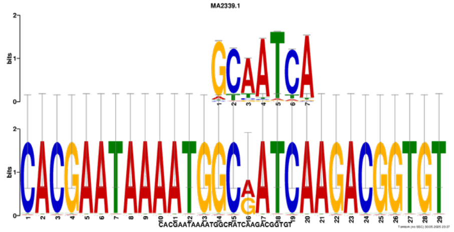

FOXB1

FOXK1

LHXB

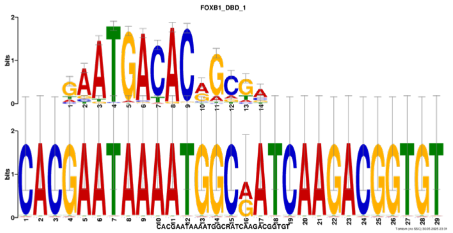

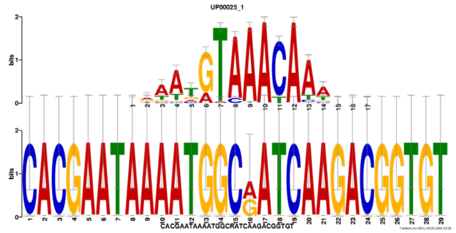

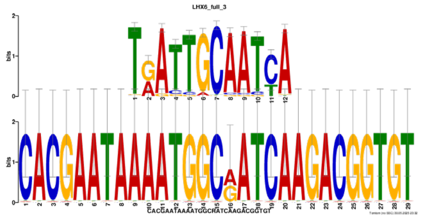

LHX8

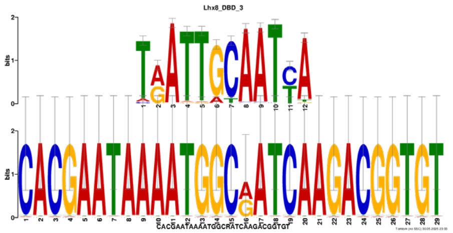

NC_010444.4:g.6757679C>T

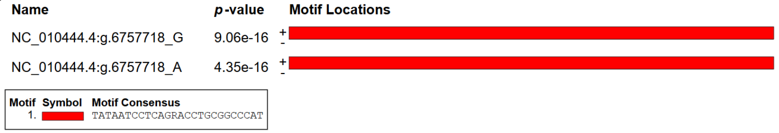

ZNF454

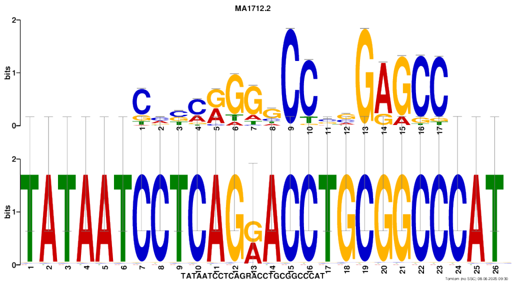

rs318806718

Name	*p*-value	Motif Locations
rs318806718_A	2.08e-15	+ -
rs318806718_G	4.96e-15	+ -

Motif	Symbol	Motif Consensus
1.		AGGGTTATTGGRTGACGGGGGTGTG

NR1D1, NR1D2

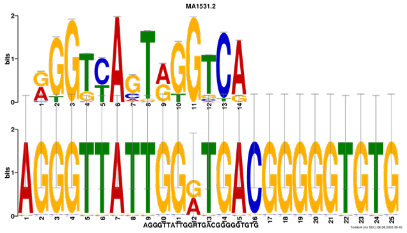

PBX3

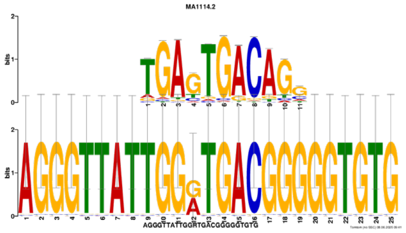

Sox17

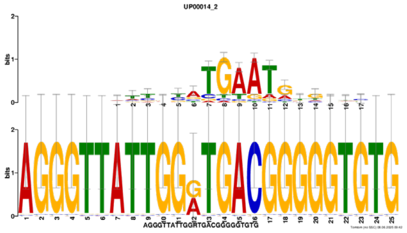

XBP1

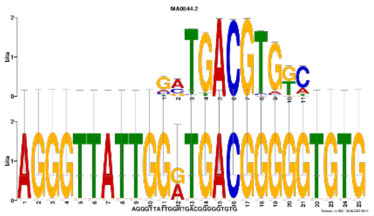

rs319966827

Name	*p*-value	Motif Locations
rs319966827_C	4.02e-12	
rs319966827_G	4.02e-12	

Motif	Symbol	Motif Consensus
1.		CTGTACTCSAAGGAAACACA

BCL6B

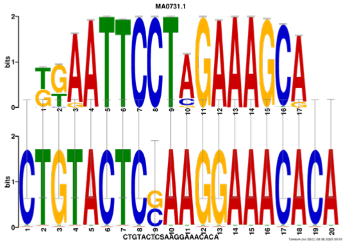

NFATC1

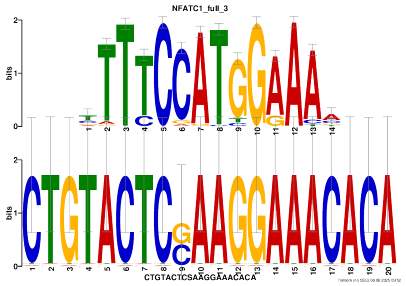

rs338175674

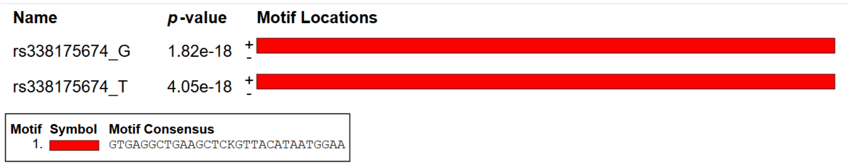

ZSCAN16

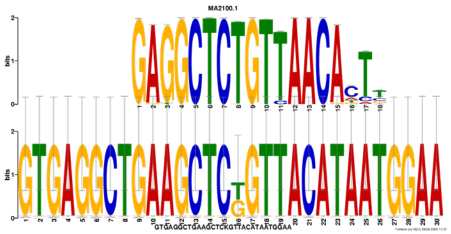

Supplement: Supplementary file 1 [file genes-17-00888-s001.zip › genes-4385819-supplementary.pdf]
